# The oral-gut-joint axis in osteoarthritis: a multiomics case-control study

**DOI:** 10.3389/fcimb.2026.1833218

**Published:** 2026-06-22

**Authors:** Yuchi Liu, Jinhua Gong, Yuying Zhang, Hongyu Wang, Haotian Feng

**Affiliations:** 1 Department of Orthopaedics, Shandong Provincial Hospital Affiliated to Shandong First Medical University, Jinan, China; 2 Qingdao University, Qingdao Medical College, Qingdao, China; 3 Department of Gastroenterology, Weifang People’s Hospital, Shandong Second Medical University, Weifang, China; 4 Department of Joint Surgery, Weifang People’s Hospital, Shandong Second Medical University, Weifang, China

**Keywords:** 16S rDNA, gastrointestinal microbiome, multi-omics research, oral-gut-joint axis, osteoarthritis, proteomics, transcriptome

## Abstract

**Background:**

Osteoarthritis (OA) is a globally prevalent degenerative joint disorder that imposes significant socioeconomic burdens. While traditionally viewed as a localized “wear-and-tear” disease, emerging evidence supports a systemic pathogenesis involving the gut-joint axis. The oral-gut-joint pathway remains underexplored in OA pathophysiology.

**Objective:**

This study aimed to characterize oral and gut microbiota signatures in OA patients and elucidate their functional connections to cartilage degeneration through multiomics integration.

**Methods:**

We conducted a cross-sectional observational study involving 25 OA patients and 20 healthy controls. 16S rDNA gene amplicon sequencing was performed on fecal and oropharyngeal swab samples. Cartilage tissues were subjected to transcriptomic and proteomic analyses.

**Results:**

We identified distinct dysbiosis patterns in both the gut and oral microbiomes of OA patients. The α-diversity of the gut microbiota significantly increased (P<0.05) with enrichment of *Ruminococcaceae* and *Subdoligranulum*. Concurrently, the oral microbiota showed increased α-diversity and activation of the lipopolysaccharide biosynthesis pathway. We constructed two significant cross-omics correlation modules: one linking gut microbes (*Lachnospiraceae* and *Muribaculaceae*) to cartilage inflammatory genes (*MAPK11*, *ITGB3*, *CD55* and *ANGPT2*) and extracellular matrix remodelling proteins and another connecting gut microbes (*Helicobacter*, *Pseudomonas*, and *Phocea*) with *CXCL14* and *GNGT2*.

**Conclusion:**

Our study revealed the dysbiotic characteristics of the oral-gut microbiome and its complex associations with pathological changes in cartilage. These findings offer novel mechanistic insights and potential therapeutic targets for microbiota-based precision interventions in OA.

## 1 Introduction

Osteoarthritis (OA), a chronic degenerative joint disease characterized by synovitis, cartilage degradation, bone remodeling, and osteophyte formation, is a leading global cause of pain and disability (Martel-Pelletier et al., 2016). A 2020 Global Burden of Disease study reported 595 million OA cases (7.6% of the global population), a number projected to increase exponentially by 2050 (Hunter and Bierma-Zeinstra, 2019; G.O. Collaborators, 2023). Traditionally regarded as a localized “wear-and-tear” condition driven by mechanical stress and aging, OA is increasingly recognized as having a systemic pathogenesis. Many OA patients without significant joint injury exhibit persistent low-grade systemic inflammation (Robinson et al., 2016), along with metabolic dysfunction and the activity of adipose tissue-derived inflammatory mediators (Binvignat et al., 2024; Herrero-Beaumont et al., 2024). Understanding the metabolic drivers of systemic inflammation has become essential for developing more effective therapeutic strategies.

As the most metabolically complex organ in the human body, the gut microbiota generates bioactive metabolites capable of regulating host physiology across multiple systemic axes, such as the gut-bone pathways (Borody and Campbell, 2012; Dong et al., 2025). Clinical observations indicate that the microbial community differs significantly in patients with knee OA, showing reduced abundance of short-chain fatty acid producers (e.g., Faecalibacterium and Lachnospiraceae) and expansion of proinflammatory Enterobacteriaceae (Sánchez Romero et al., 2021; Ning et al., 2022). This dysbiosis not only alters the local immune microenvironment but also remotely reshapes joint immunity through circulating metabolites (Sun et al., 2025). For example, studies in animal models have demonstrated that gut microbiome dysbiosis may correlate with OA progression by inducing infrapatellar fat pad-synovial membrane inflammation, and that increased gut permeability and intestinal inflammation may precede the onset of arthritis (Hecquet et al., 2023; Liu et al., 2025). Specific gut commensals, such as *Faecalibacterium prausnitzii*, have been shown to alleviate inflammatory arthritis by modulating the production of IL-17 and short-chain fatty acids (Moon et al., 2023). Lipopolysaccharide (LPS) activates synovial macrophage M1 polarization through the TLR4-MyD88 pathway, creating a proinflammatory feedback loop (Huang et al., 2020; Liu et al., 2023). Butyrate promotes peripheral Treg differentiation to suppress inflammation, and secondary bile acids regulate chondrocyte survival through vitamin D receptor signaling (Boer et al., 2019; Liu et al., 2019).

The oral microbiota, another potential inflammatory driver, has been underexplored in OA research. As the second most microbially dense human site, the oral cavity continuously interacts with the gut microbiota (Catalan et al., 2024). A large cohort study revealed a significant bidirectional relationship between periodontitis and OA in more than 140,000 patients (Ma et al., 2022). Mechanistically, oral commensals such as *Fusobacterium nucleatum* can secrete outer membrane vesicles that alter gut immune homeostasis upon ingestion and may be linked to OA (Hong et al., 2023). Products of oral pathogens can enter the bloodstream and induce systemic inflammation (Yamazaki and Kamada, 2024). This “oral-gut-joint” axis suggests that studying the gut or oral microbiota in isolation may not fully capture the microbial signature of OA.

Based on emerging evidence for the oral-gut-joint axis, we hypothesized that OA patients exhibit distinct oral-gut dysbiosis functionally associated with cartilage molecular changes. This study aims to map the relationship between oral-gut microbiota dysbiosis and joint degeneration in OA patients through multiomics integration, offering new experimental evidence for the systemic mechanisms of OA.

## 2 Results

### 2.1 Study workflow and patient clinical information

**Figure 1:**
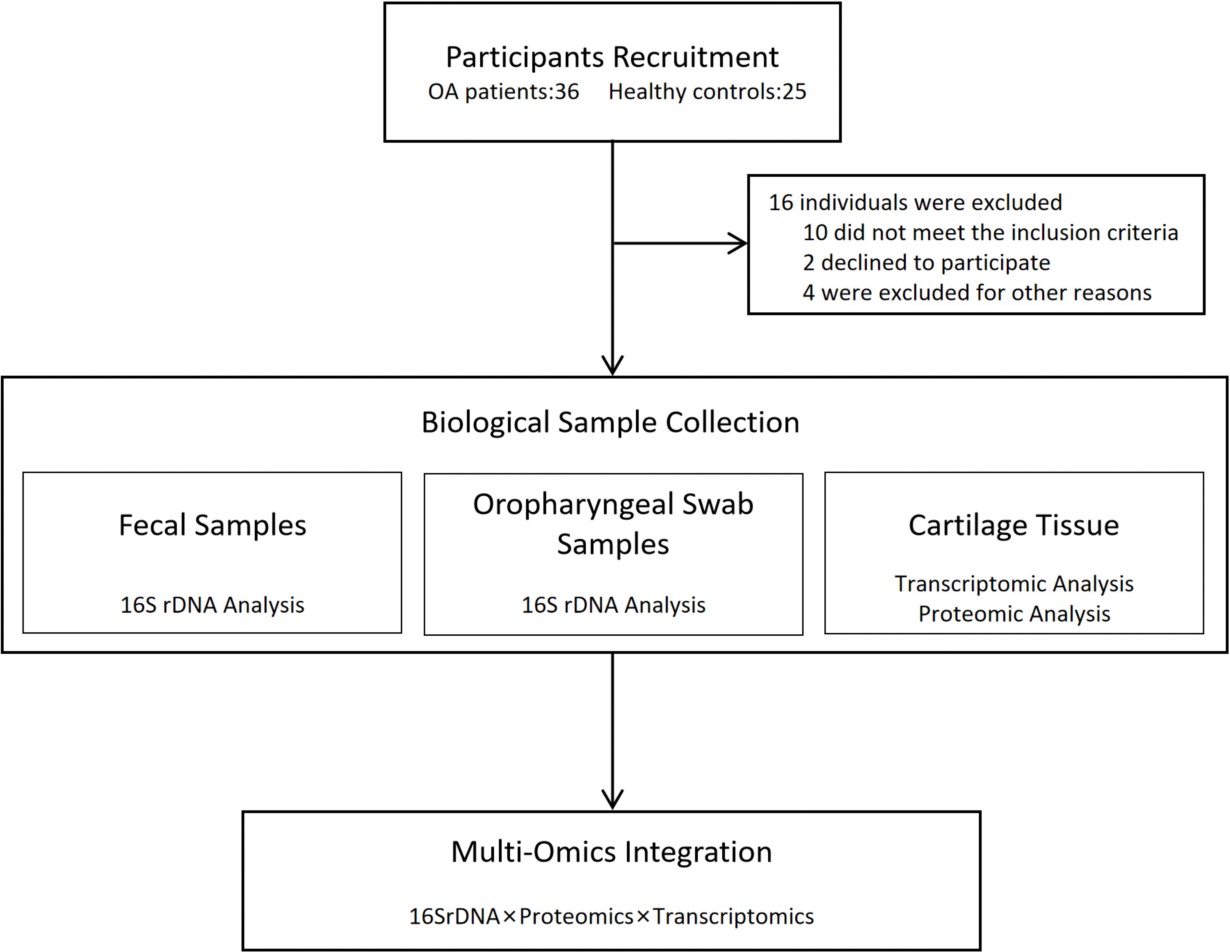
Study workflow.

In this study, 36 OA patients and 25 healthy controls (HC) were included, with 45 participants (25 OA patients and 20 matched HCs) ultimately included for multiomics analysis. OA patients were visited and recruited at Weifang People’s Hospital between July 1, 2025, and October 15, 2025, and HCs were community-recruited. The experimental flowchart is shown in **Figure 1**. Baseline demographic data for the participants are provided in **Supplementary Materials S1**. Potential confounding factors, including body mass index, smoking status and drinking status, were collected. Potential confounders were considered in downstream analyses where appropriate, or their impact was acknowledged as a limitation. There were no significant differences in age (OA: 66.6 ± 7.9 vs. HC: 67.5 ± 6.0 years; p = 0.653), sex distribution (OA: 52% female vs. HC: 40% female; p = 0.616) and body mass index (OA: 27.64 ± 2.8 vs. HC: 26.27 ± 2.25; p = 0.076) between the two groups, confirming their comparability. Clinical samples (including cartilage from inflammatory and noninflammatory regions in OA patients and blood, feces, and oral swabs from all participants) were used for subsequent gut/oral microbiota analysis and cartilage transcriptomics/proteomics.

### 2.2 Gut and oral microbiota dysbiosis in OA patients

**Figure 2:**
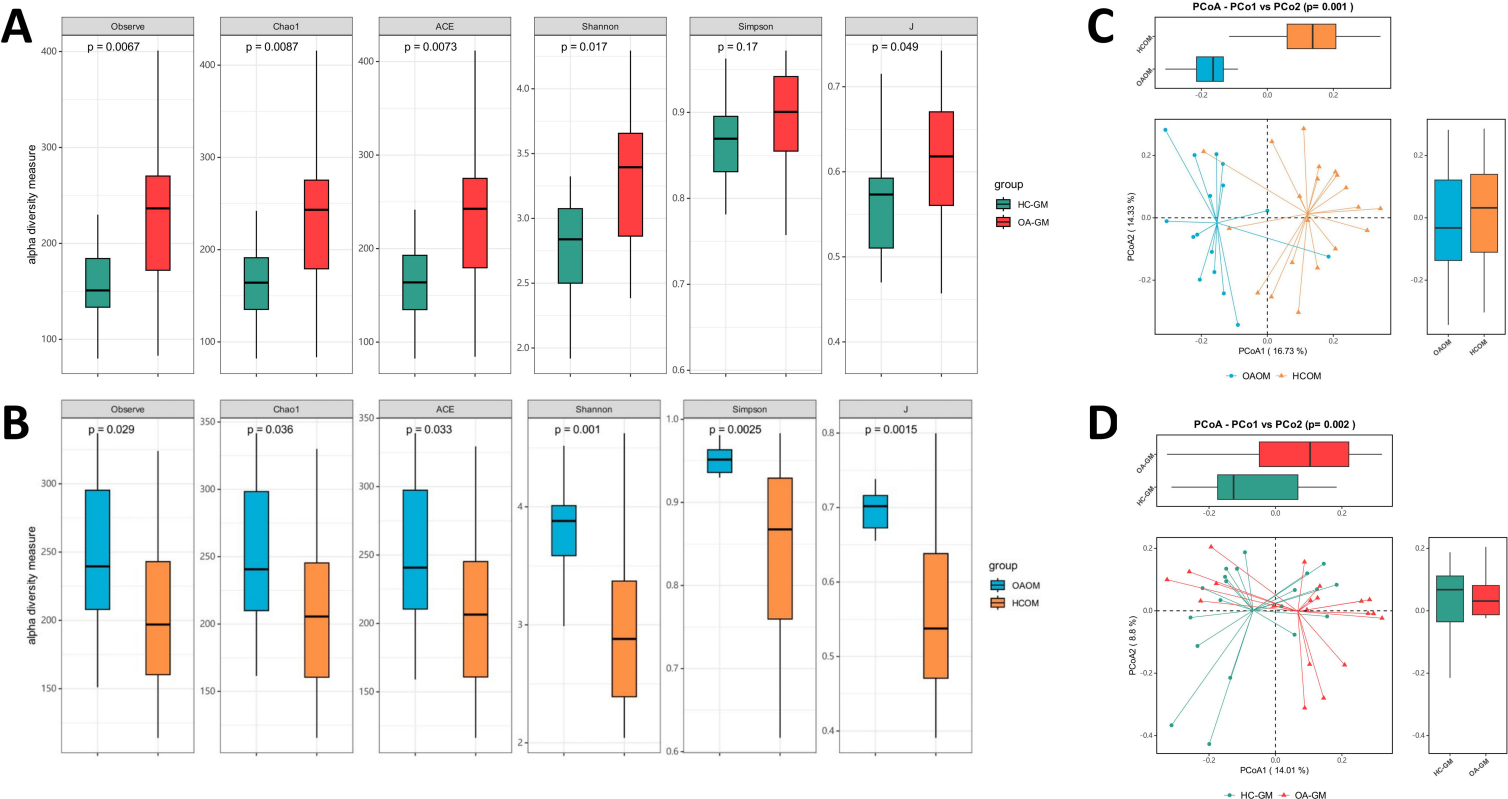
Altered diversity and composition of the gut and oral microbiota in OA patients. **(A)** α-diversity indices of the gut microbiota, including observed species and Chao1, ACE, J, and Shannon indices (Wilcoxon rank-sum test, P < 0.05). **(B)** α-diversity indices of oral microbiota, including observed species and Chao1, ACE, Shannon, J, and Simpson indices (Wilcoxon rank-sum test, P < 0.05). **(C, D)** Principal coordinate analysis (PCoA) based on the weighted UniFrac distance of the gut **(C)** and oral **(D)** microbial communities between OA patients and HCs (PERMANOVA, P = 0.001 and P = 0.002).

To explore the microbial characteristics of OA patients, 16S rDNA gene sequencing was performed on fecal and oral samples from OA patients and HCs. α-diversity analysis revealed that compared with HCs, OA patients had significantly greater observed, Chao1, ACE, J and Shannon indices (p < 0.05), with no significant differences in Simpson (**Figure 2A**). These findings indicated a significant increase in species richness in the gut microbiota of OA patients. However, the concentrations of the dominant bacteria in the two groups were similar. With respect to the oral microbiota, all α-diversity indices were significantly greater in OA patients than in healthy individuals (**Figure 2B**). β-diversity, such as PCoA, clearly distinguished the gut and oral microbiota between OA patients and HCs (p = 0.001, p = 0.002) (**Figures 2C, D**).

**Figure 3:**
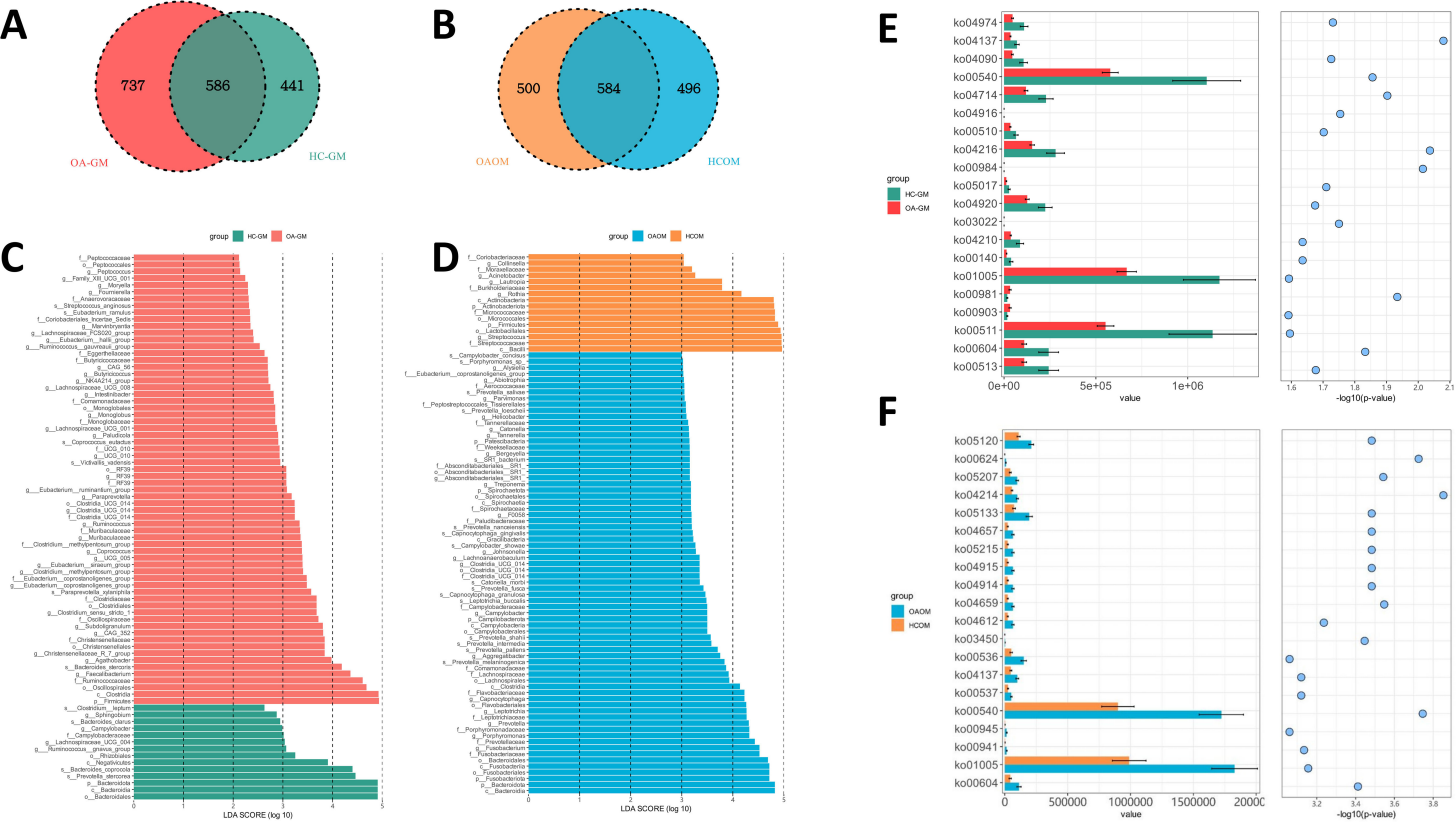
Taxonomic and functional characteristics of the gut and oral microbiota in OA. **(A)** Venn diagram of the differences in the gut microbiota between OA patients and HCs. **(B)** Venn diagram of the oral microbiota between OA patients and HCs. **(C)** LEfSe analysis of differentially abundant taxa (LDA score > 2, P < 0.05) in the gut microbiota. **(D)** LEfSe analysis of differentially abundant taxa (LDA score > 2, P < 0.05) in the oral microbiota. **(E)** PICRUSt2 functional prediction of the gut microbiota at the KEGG pathway level. **(F)** PICRUSt2 functional prediction of oral microbiota at the KEGG pathway level.

At the community composition level, Venn diagram of the gut microbiota revealed586 operational taxonomic units (OTUs)shared between OA patients and HCs, with 737 unique to OA patients and 441 unique to HCs (**Figure 3A**). Similar patterns were observed for the oral microbiota, with 584 shared OTUs, 500 unique to OA patients, and 496 unique to HCs (**Figure 3B**). LEfSe analysis revealed differentially abundant taxa between the two groups. In the gut microbiota, OA patients were enriched in f_Ruminococcaceae, s_un_g_Subdoligranulum, g_Subdoligranulum, etc. While HCs had higher abundances of c_Negativicutes, o_Selenomonadales, s_un_g_Megamonas, etc. (**Figure 3C**). In the oral microbiota, OA patients were enriched in c:Bacteroidia, p_Bacteroidota and p_Fusobacteriota, etc. Whereas HCs were enriched in c_Bacilli, f_Streptococcaceae and g_Streptococcus, etc. (**Figure 3D**).

PICRUSt2-based functional prediction revealed significant differences in metabolic pathways of the gut and oral microbiota between OA patients and HCs at the KEGG pathway level. In terms of the gut microbiota, OA patients differed significantly from HCs in terms of pathways such as apoptosis (**Figure 3E**). In terms of oral microbiota, OA patients showed predicted enrichment of pathways such as LPS biosynthesis and polycyclic aromatic hydrocarbon degradation (**Figure 3F**).

### 2.3 Cartilage transcriptomic and proteomic profiles

**Figure 4:**
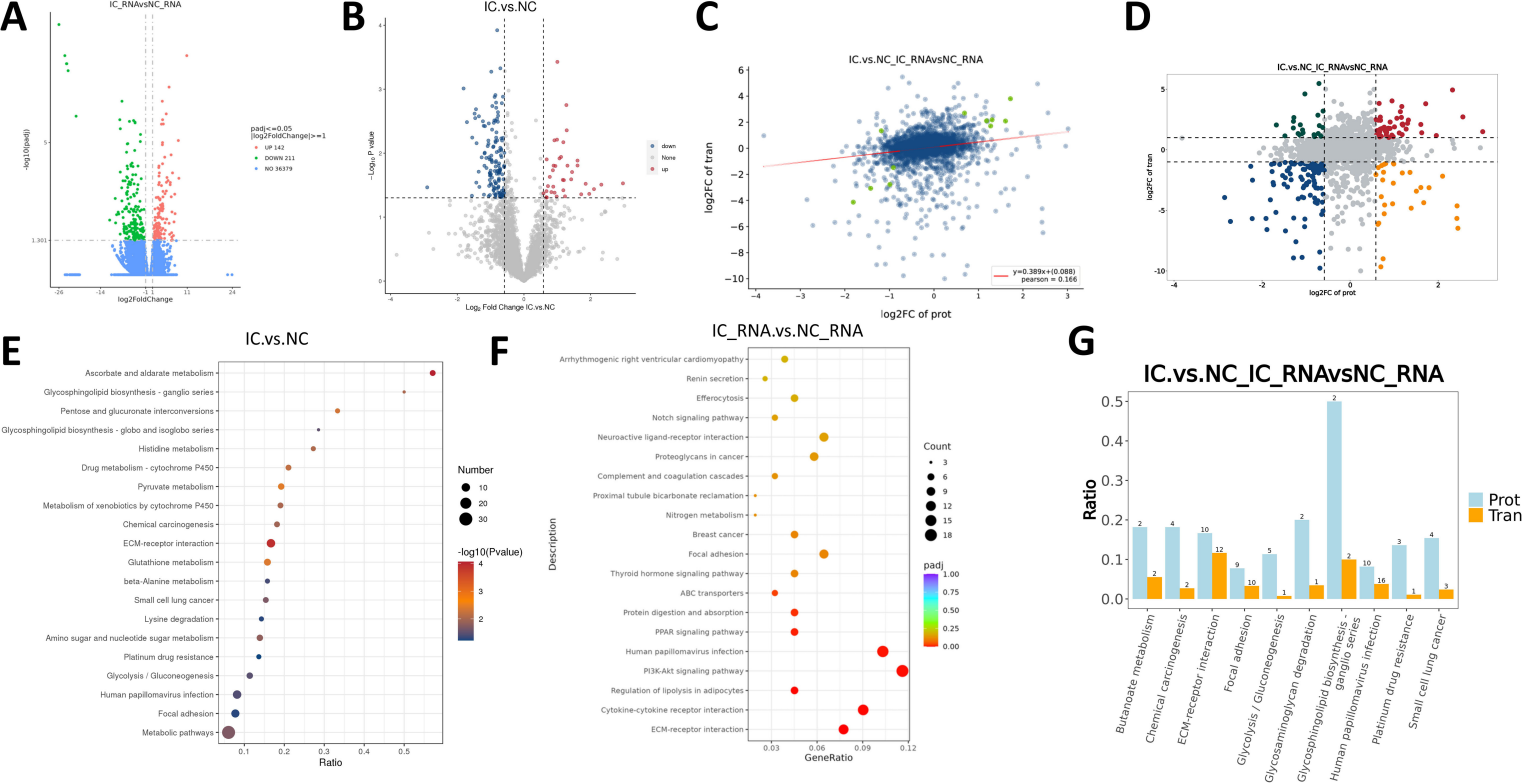
Transcriptomic and proteomic profiling of inflammatory versus noninflammatory cartilage regions in OA patients. **(A)** Volcano plot of DEGs identified by DESeq2 (|log_2_FoldChange| ≥ 1, adjusted P ≤ 0.05). **(B)** Volcano plot of DEPs identified by DIA-NN (fold change > 1.5, P ≤ 0.05). **(C)** Correlation analysis between mRNA and protein expression levels for commonly detected genes/proteins (Pearson correlation). **(D)** Nine-quadrant plot showing the joint distribution of transcriptomic and proteomic changes. **(E)** KEGG pathway enrichment analysis of the DEPs. **(F)** KEGG pathway enrichment analysis of DEGs. **(G)** Distribution comparison of enriched KEGG pathways between DEPs and DEGs.

We performed transcriptomic analysis and proteomic analysis on paired cartilage samples from inflammatory and noninflammatory regions from OA patients. We performed differential expression analysis on transcriptional data from inflamed and noninflamed cartilage tissues of OA patients using DESeq2 [|log2(FoldChange)| ≥ 1, adjusted P ≤ 0.05]. A total of 353 differentially expressed genes (DEGs) were identified, including 142 upregulated genes and 211 downregulated genes (**Figure 4A**). The volcano plot shows a scattered distribution of DEGs between the two groups, indicating extensive and complex transcriptional changes under OA inflammation. Proteomic analysis using DIA-NN (fold change > 1.5, P ≤ 0.05) revealed 177 differentially expressed proteins (DEPs), 37 of which were upregulated and 140 of which were downregulated (**Figure 4B**). To assess the consistency between the transcriptomic and proteomic data, we conducted correlation analysis on commonly identified genes/proteins. The results revealed a weak positive correlation between protein expression and gene expression (Pearson = 0.166) (**Figure 4C**). A nine-quadrant plot was constructed for the joint analysis of the DEPs and DEGs (**Figure 4D**).

KEGG pathway enrichment analysis of the DEPs (**Figure 4E**) revealed significant pathways such as extracellular matrix (ECM)-receptor interaction, ascorbate and aldarate metabolism, pyruvate metabolism, and glutathione metabolism, suggesting pronounced ECM degradation, oxidative stress, and metabolic dysfunction in inflamed OA regions. KEGG analysis of the DEGs (**Figure 4F**) revealed significant pathways related to ECM-receptor interactions, cytokine-cytokine receptor interactions, the PI3K-Akt signaling pathway, and the regulation of lipolysis in adipocytes. Notably, inflammatory pathways were more significantly enriched in the transcriptomic data, whereas metabolic pathways were more strongly enriched in the proteomic data. A comparison of KEGG pathway enrichment between the proteomic and transcriptomic data (**Figure 4G**) revealed that metabolic pathways (glycosphingolipid biosynthesis and glycosaminoglycan degradation) were substantially enriched at the protein level, whereas the enrichment of ECM-receptor interactions was relatively balanced in both datasets.

### 2.4 Integrative analysis of gut microbiota-cartilage interactions

**Figure 5:**
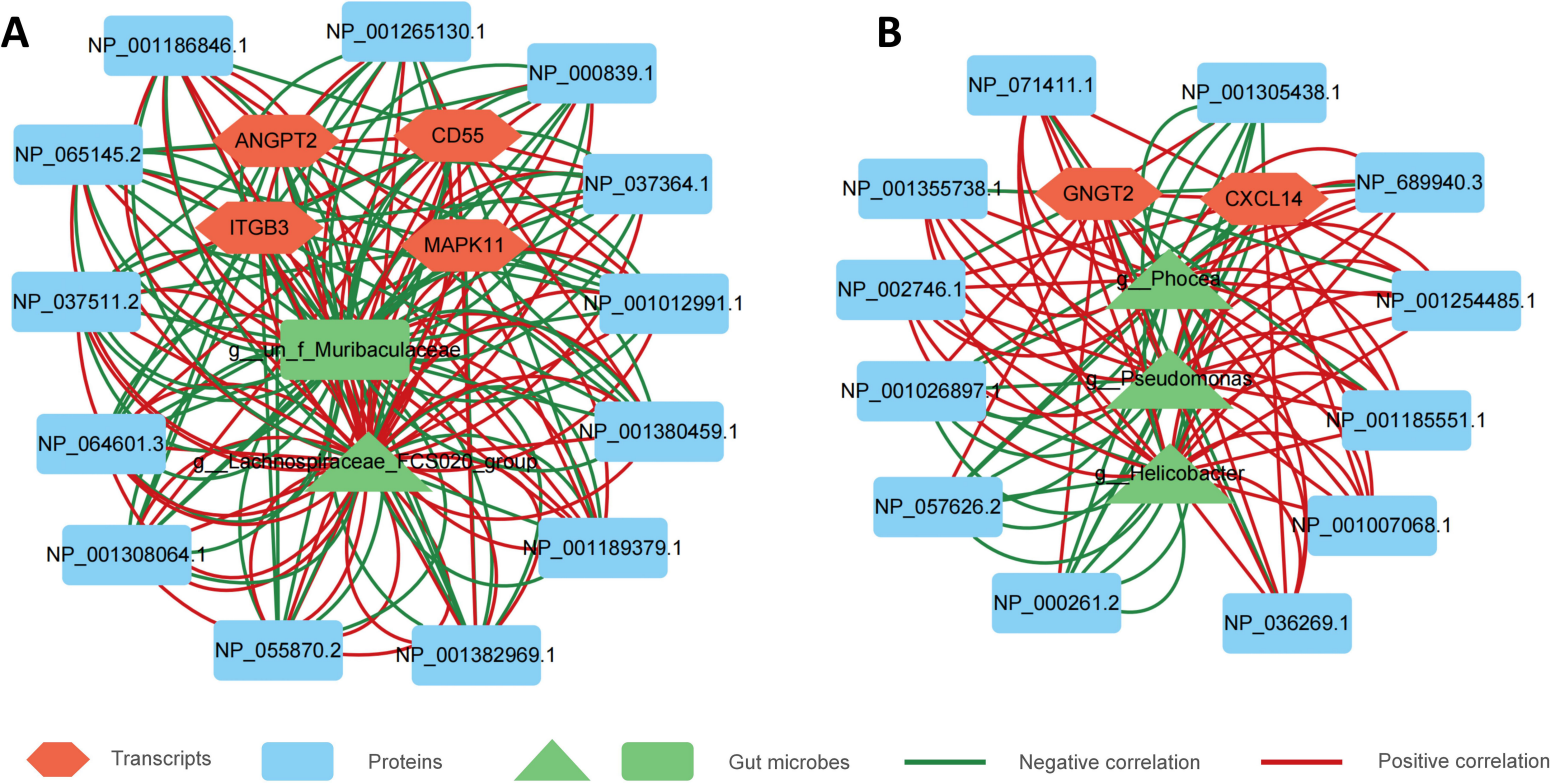
Integration of the gut microbiota with cartilage transcriptomic and proteomic profiles in OA patients. **(A)** Associations of g_*Lachnospiraceae*_FCS020_group and g_un_f_*Muribaculaceae* with cartilage genes and proteins. **(B)** Associations of g_*Helicobacter*, g_*Pseudomonas and* g_*Phocea* with cartilage genes and proteins.

We performed an integrated analysis of the gut microbiota, cartilage transcriptome, and proteome in OA patients. By constructing a triomic correlation network, we identified two significantly correlated communities. The first community revealed significant correlations between the gut microbes g_*Lachnospiraceae*_FCS020_group and g_un_f_*Muribaculaceae* and the expression levels of genes (*MAPK11*, *ITGB3*, *CD55*, *ANGPT2*) and proteins (phosphoacetylglucosamine mutase isoform 1, etc.). g_*Lachnospiraceae*_FCS020_group was negatively correlated with *MAPK11* and *ITGB3* expression, whereas g_un_f_*Muribaculaceae*was positively correlated with both genes. Genes such as *MAPK11* are involved in cellular stress, inflammation, differentiation, angiogenesis, and autoimmunity, whereas proteins such as NP_001186846.1 are linked to cell signaling, adhesion, and angiogenesis (**Figure 5A**). The second community linked gut microbes (g_*Helicobacter*, g_*Pseudomonas*, and g_*Phocea*) with cartilage transcriptome genes (*CXCL14, GNGT2*) and proteome proteins (acyl-coA synthetase long chain family member 4). All these elements showed significant pairwise correlations. These genes were negatively correlated with *GNGT2* and positively correlated with *CXCL14* (**Figure 5B**). These genes play key roles in inflammation and cell communication, suggesting that these microbes may play a role in OA inflammation by regulating their expression. Proteins such as NP_001305438.1 (long-chain-fatty-acid-CoA ligase 4 isoform 2) are involved in inflammation and ECM remodeling, indicating potential microbial impacts on cartilage inflammation and degeneration.

## 3 Discussion

We identified distinct dysbiosis patterns in the gut and oral microbiota of OA patients. These multi-layered data support the existence of an “oral-gut-joint axis”, tentatively connecting specific gut microbes to cartilage inflammatory genes and matrix remodeling proteins.

Our study revealed that the α-diversity of the gut microbiota in OA patients was significantly greater than that in healthy controls, as reflected by increased richness and evenness indices. The unchanged Simpson diversity might suggest that this elevation derives from the expansion of rare taxa rather than dominant community restructuring. These findings differ from those of previous studies reporting reduced gut microbiota diversity in OA patients (Liu et al., 2022). We suspect this discrepancy stems from various factors, including patient geographical location, dietary habits, disease staging, comorbidities, and sequencing technologies (Yatsunenko et al., 2012; Falony et al., 2016). Geographically, our cohort was recruited from northern China, where dietary patterns may shape distinct gut microbial profiles compared to Western or southern Chinese populations. However, an increase in microbial diversity is not always indicative of health; it may reflect a decline in gut ecological stability and the opening of ecological niches, thereby providing opportunities for the enrichment of potential pathogens (Yao et al., 2024). Significant β-diversity separation indicates reproducible, disease-associated community restructuring between OA patients and healthy controls. β-diversity captures between-group compositional dissimilarity, and its divergence in our cohort suggests that OA is characterized by a consistent microbial community signature rather than random variation. The 737 unique OTUs identified in OA patients in this study might include more opportunistic pathogens with proinflammatory potential. At the taxonomic level, we observed enrichment of *Ruminococcaceae* in the gut of OA patients. Notably, members of the *Ruminococcaceae* family exhibit extremely high functional heterogeneity (Wang et al., 2023); some are major butyrate producers, whereas others are associated with gut inflammation and the presence of LPS (Joossens et al., 2011; Davis et al., 2021; Milton-Laskibar et al., 2022; Han et al., 2025).

Our study also revealed significant alterations in the oral microbiome of OA patients, characterized by a comprehensive increase in α-diversity and significant enrichment of the LPS biosynthesis pathway. As the second most microbially dense site in the human body, the oral cavity continuously interacts with the gut microbiota (Yeo et al., 2022). Research has confirmed a significant bidirectional relationship between periodontitis and OA (Ma et al., 2022). Mechanistically, oral pathobionts (e.g., *Porphyromonas gingivalis*) and their products can migrate to the gut via the “oral-gut-joint” axis, disrupting gut immune homeostasis (Lorenzo et al., 2019), and can also activate systemic inflammatory responses (de Oliveira et al., 2022; Yamazaki and Kamada, 2024). Nanovesicles carrying *Porphyromonas gingivalis* antigens have been detected in the plasma of periodontitis patients, providing direct evidence for the entry of oral pathogens and their products into systemic circulation (Farrugia et al., 2020; Wang et al., 2022). The predicted enrichment of the LPS biosynthesis pathway in the oral microbiota in this study further supports the possibility that orally derived LPS may be linked to OA inflammation through systemic routes.

In this study, differential expression analysis of transcriptomic and proteomic data from inflamed and noninflamed cartilage tissues from OA patients was performed. Proteomic data highlighted significant pathways such as ECM-receptor interaction, ascorbate and aldarate metabolism, and glutathione metabolism. The enrichment of the ECM-receptor interaction pathway suggests abnormal degradation and remodeling of the ECM in OA cartilage, which is consistent with characteristic cartilage damage in OA (Hao et al., 2021; Liu et al., 2022; Moore et al., 2022). Changes in glutathione metabolism point to the important role of oxidative stress in OA, which is considered a key driver of chondrocyte damage and apoptosis through the production of reactive oxygen species that impair cellular structure and function (Zhu et al., 2020; Zhao et al., 2023). Transcriptomic data, on the other hand, revealed inflammation-related pathways, including cytokine-cytokine receptor interactions, the PI3K-Akt signaling pathway, and the regulation of lipolysis in adipocytes. Upon binding to receptors on the cell membrane, many cytokines (e.g., interleukin-1β, tumor necrosis factor-α, and interleukin-6) activate the NF-κB signaling pathway through the PI3K/Akt and MAPK signaling pathways, thereby affecting OA (Lee et al., 2022). Integrated analysis revealed that ECM-receptor interaction and focal adhesion pathways were highly enriched in both the transcriptomic and proteomic datasets. These findings are highly consistent with reports in the literature (Sun et al., 2024) and provide a multilevel molecular perspective for understanding OA. The weak transcriptome-proteome correlation (Pearson = 0.166) may reflect several biological and technical factors. Post-transcriptional regulatory mechanisms, including microRNA-mediated mRNA degradation, variable translational efficiency, protein turnover dynamics, and post-translational modifications, commonly weaken the correspondence between mRNA and protein levels. Additionally, sample heterogeneity in cartilage tissue composition, such as variable chondrocyte density, extracellular matrix content, and inflammatory cell infiltration, may contribute to this divergence. These considerations suggest that transcriptomics and proteomics capture distinct yet complementary molecular layers, and their integration remains valuable for understanding OA cartilage pathophysiology.

In the constructed “microbiota-gene-protein” coexpression network, two significant correlation modules were identified. The first module linked the gut bacteria *Lachnospiraceae* and *Muribaculaceae* with the expression levels of genes (*MAPK11*, *ITGB3*, *CD55*, *ANGPT2*) and proteins (NP_001186846.1). The activation of *MAPK11* (p38β), a member of the p38 MAPK family, is closely related to chondrocyte apoptosis and matrix metalloproteinase 13 expression, potentially serving as an important signaling pathway for OA inflammation and cartilage degradation (Zheng et al., 2024; Meng et al., 2025). *ITGB3*, a member of the integrin family, plays a crucial role in mechanosignal transduction between cells and the ECM; functional abnormalities in this gene may affect the response of chondrocytes to mechanical stress and ECM maintenance (Li et al., 2023; Lian et al., 2025). Although both *Lachnospiraceae* and *Muribaculaceae* are important short-chain fatty acid producers, *Lachnospiraceae* was negatively correlated with *MAPK11* and *ITGB3*, whereas *Muribaculaceae* was positively correlated with *MAPK11* and *ITGB3*. These findings raise the hypothesis that *Lachnospiraceae* and *Muribaculaceae* may be associated with chondrocyte inflammation through other metabolites (e.g., branched-chain fatty acids and amines), outer membrane vesicles, or other signaling molecules. The second module linked the gut microbes *Helicobacter*, *Pseudomonas*, and *Phocea* with cartilage transcriptome genes (*CXCL14* and *GNGT2*) and proteome proteins (NP_001305438.1, etc.). *Helicobacter* and *Pseudomonas* are generally considered potential pathogens (Raman et al., 2018; Duan et al., 2025); their overgrowth in the gut may lead to impaired intestinal barrier function, promoting the entry of bacterial products such as LPS into the systemic circulation and triggering chronic low-grade systemic inflammation (Ning et al., 2024; Sun et al., 2025). This systemic inflammation is considered a significant driving factor in OA pathogenesis, exacerbating cartilage degradation and synovial inflammation (Sun et al., 2023). *CXCL14* is a chemokine that is involved primarily in immune cell recruitment and inflammatory responses (Gowhari Shabgah et al., 2021). The positive correlation of *Helicobacter* and *Pseudomonas* with *CXCL14* may indicate that these bacteria activate immune responses, leading to the upregulation of *CXCL14* expression, thereby promoting the infiltration of inflammatory cells into the joint area and exacerbating the inflammatory process of OA.

Our study links oral-gut microbiome dysbiosis to OA cartilage degeneration. However, we must admit that the modest sample size limits the robustness of our multiomics correlation networks. The limited statistical power may increase false positive risks. The networks in **Figure 5** should be interpreted as exploratory hypotheses requiring validation in larger cohorts. Additionally, this study did not conduct subgroup analyses for females and males. The cross-sectional design of this study prevents the determination of causality between dysbiosis and OA progression, and the sample size limits the statistical power of subgroup analyses. Furthermore, direct evidence for further mechanistic research is lacking; thus, cellular and animal experiments should be conducted in the future to further clarify the underlying mechanisms involved.

From a clinical perspective, our findings suggest that the oral-gut-joint axis may represent a modifiable therapeutic target in OA. The identification of reproducible microbial signatures and their correlation with cartilage inflammatory markers provides a rationale for future interventional studies, including microbiome-based biomarker development for risk stratification, targeted microbiome modulation as adjunctive therapy, and oral health management as a component of comprehensive OA care.

## 4 Materials and methods

### 4.1 Study design and participants

This cross-sectional case-control observational study included participants from Weifang People’s Hospital and the community between July 1, 2025, and October 15, 2025. A total of 45 participants (25 patients with knee OA and 20 matched HCs) were included in the multiomics analysis. The study was approved by the Medical Research Ethics Committee of Weifang People’s Hospital on October 23, 2024 (Approval No: KYLL20241023-1) and registered with the Chinese Clinical Trial Registry on June 23, 2025 (Registration number: ChiCTR2500104749). Written informed consent was obtained from all participants.

OA patients were diagnosed according to the Chinese Guidelines for the Diagnosis and Treatment of Osteoarthritis (2021 Edition), fulfilling clinical and radiographic criteria (Kellgren-Lawrence grade ≥ II) and experiencing active symptoms (mean pain score ≥ 4 on a 0–10 numeric rating scale over the past week) with a disease duration of at least 6 months. HCs had no clinical diagnosis of OA, rheumatoid arthritis, gout, or other systemic inflammatory joint diseases and reported no chronic knee pain, stiffness, or low back pain requiring medical attention during the past year.

The inclusion criteria for all participants included age over 18 years (no sex restrictions), willingness to cooperate, and provision of informed consent. The exclusion criteria included other serious diseases (e.g., rheumatoid arthritis or gout), active infection or severe infection within the past three months, immunosuppression or use of immunosuppressants, joint surgery within the past six months, current use of medications affecting joint inflammation (e.g., long-term high-dose steroids), pregnancy or breastfeeding, and severe mental disorders. To minimize potential biases, HCs were community-recruited and matched with OA patients for age and sex. Standardized protocols were employed for sample collection and laboratory analysis to reduce information bias.

For continuous variables including age and BMI, we employed independent samples t-tests when normality assumptions were met (assessed via Shapiro-Wilk testing), otherwise using Mann-Whitney U tests. Categorical variables were analyzed using Chi-square tests or Fisher’s exact probability tests where expected cell counts were low.

### 4.2 Sample collection and storage

All laboratory procedures were conducted at the Central Laboratory of Weifang People’s Hospital. Fecal samples were self-collected using sterile containers. Oral samples were collected from the posterior oropharynx using sterile swabs. Blood was collected in EDTA tubes. Discarded cartilage tissues were collected during surgery, rapidly frozen in liquid nitrogen, and stored at -80 °C. All the samples were transported on ice to the laboratory within 2 hours. Fecal and oral samples were stored at -80 °C until DNA extraction. Plasma was obtained from blood by centrifugation (3,000×g for 10 min), aliquoted, and stored at -80 °C. The tissue samples were kept at -80 °C until analysis.

### 4.3 16S rDNA gene sequencing and analysis

Microbial DNA was extracted using a QIAamp DNA Fecal Mini Kit. The V3-V4 hypervariable region was amplified by PCR using forward (5’-CCTACGGGNBGCASCAG-3’) and reverse (5’-GGACTACNVGGGTWTCTAAT-3’) primers. PCR products were purified and subjected to paired-end sequencing on an Illumina HiSeq 2500 platform.

Raw paired-end reads were assembled using FLASH (Magoč and Salzberg, 2011). Primers and low-quality reads were removed with Cutadapt (Martin, 2011). Chimera checking and OTU clustering were performed using usearch (Edgar, 2010). Representative OTU sequences were aligned against the silva_132_97_16S (DeSantis et al., 2006) database for taxonomic classification via the RDP (Cole et al., 2014) Classifier. Phylogenetic trees were constructed using FastTree (Price et al., 2009).

Functional prediction was performed using PICRUSt (Langille et al., 2013) to infer metagenomic function content from 16S data. α-diversity (Observed richness, Shannon, Simpson, ACE, Chao1, Pielou’s evenness (J’)) and β-diversity (Bray-Curtis distance) were calculated using the R package vegan (Oksanen et al., 2013). Statistical significance for α-diversity differences was determined by a nonparametric Kruskal-Wallis rank sum test with Benjamini-Hochberg correction. β-diversity differences were assessed using PERMANOVA with 999 permutations in the R package vegan (Oksanen et al., 2013). The LEfSe (Paulson et al., 2013) analysis was performed to identify taxa with differentiating abundance in the different group. LEfSe (Paulson et al., 2013) is an algorithm for high-dimensional biomarker discovery and explanation that identifies genomic features characterizing the differences between two of more biological conditions.

### 4.4 Proteomic analysis of cartilage

Cartilage tissue was ground in liquid nitrogen and lysed with SDT buffer. Lysates underwent centrifugation, heating, alkylation, and acetone precipitation. Protein pellets were dissolved in DB buffer and digested with trypsin (Wiśniewski et al., 2009). Peptides were desalted using C18 StageTips and separated on a PepMap™ Neo C18 column. MS analysis was performed on an Orbitrap Astral mass spectrometer with an Easy-Spray ion source. Mass spectrometry data were acquired in data-independent acquisition (DIA) mode, with a full MS scan range of m/z 380–980 and a resolution of 240,000. DIA settings included 300 windows of 2 Th, NCE 25%, MS2 range m/z 150-2000, and Astral resolution 80,000. Protein identification and quantification were performed using DIA-NN software (Demichev et al., 2020) against a protein database. DEPs were defined as those with a fold change greater or less than a specified value. Functional analysis included GO and KEGG enrichmentanalyses (Huang da et al., 2009; Franceschini et al., 2013). Detailed protocols and quality control data are provided in the **Supplementary Materials S2**.

### 4.5 Transcriptomic analysis of cartilage

Total RNA was extracted using TRIzol^®^. RNA integrity was verified on an Agilent Bioanalyzer. Libraries were prepared using the NEBNext^®^ Ultra™ II Directional RNA Library Prep Kit and sequenced on an Illumina NovaSeq 6000 platform (150 bp paired-end). Clean reads were aligned to the human reference genome (GRCh38.p14) using HISAT2 (v2.2.1). Ribosomal RNA was depleted from total RNA. Transcript assembly and quantification were performed with StringTie (v2.2.3). Gene expression was calculated as FPKM values using featureCounts (v2.0.6). Differential expression analysis was conducted using DESeq2 (v1.42.0), which implements the Benjamini-Hochberg false discovery rate (FDR) method for multiple testing correction via the independent hypothesis weighting approach. Genes with adjusted p-values (padj) ≤ 0.05 and |log2FoldChange| ≥ 1 were considered significantly differentially expressed. GO and KEGG enrichment analyses were performed using clusterProfiler (4.8.1), with corrected P values less than 0.05 considered significant. Detailed protocols and quality control data are provided in the **Supplementary Materials S2**.

### 4.6 Integrated multiomics analysis

Pairwise correlation analysis between microbial, gene expression, and protein data was performed using the R package Hmisc. Prior to analysis, each omics data type was preprocessed with specific normalization methods: for microbiome compositional data, centered log-ratio transformation was applied using the compositions package in R to address compositional effects. For transcriptomics data (gene expression), counts were normalized using DESeq2’s variance-stabilizing transformation, followed by Z-score standardization across samples. For proteomics data, abundance values were log2-transformed and then Z-score standardized. All datasets were checked to ensure consistent sample names and order. Correlation coefficients and corresponding P-value matrices were obtained using the rcorr function. To correct for multiple testing, the Benjamini-Hochberg FDR method was applied. A “microbiota-gene-protein” interaction network was constructed by retaining pairwise associations that met the following thresholds: Spearman’s correlation coefficient |ρ| > 0.6 and FDR-adjusted P < 0.05. Only correlations meeting these criteria in all three pairwise comparisons (microbiota-gene, microbiota-protein, gene-protein) were retained for network construction. The resulting significant associations were visualized in Cytoscape software, where network layout (force-directed), node attributes (shape, size), and edge colors (positive: red; negative: blue) were adjusted to illustrate complex relationships and identify key nodes or modules.

## 5 Conclusion

Our integrated multiomics study constructed a “microbiota-gene-protein” correlation network to elucidate the dysbiotic features of the oral and gut microbiomes in OA patients and their complex functional connections to pathological changes in cartilage. The constructed “microbiota-gene-protein” correlation network revealed two significant cross-omics modules.

## Data Availability

The raw 16S rDNA sequencing data have been deposited in the NCBI SRA under BioProject accession number PRJNA1467634 and PRJNA1467632. The raw transcriptomic data have been deposited in GEO and the accession number is GSE331536. The mass spectrometry proteomics data have been deposited to the ProteomeXchange Consortium (https://proteomecentral.proteomexchange.org) via the iProX partner repository (Ma et al., 2019; Chen et al., 2022) with the dataset identifier PXD078593.
