# Supplementary material for: The oral-gut-joint axis in osteoarthritis: a multiomics case-control study
Source: Front Cell Infect Microbiol. 2026 Jun 22;16:1833218. doi: 10.3389/fcimb.2026.1833218 (PMC13333612; doi:10.3389/fcimb.2026.1833218)
Supplement: Supplementary file 1 [file DataSheet1.pdf]

1. Proteomic Analysis

1.1. Total Protein Extraction[1-3]

Sample was ground individually in liquid nitrogen and lysed with SDT (containing 100 mM Nacl) and 1/100 volume of DTT, followed by 5 min of ultrasonication on ice. Centrifuge at 12,000 g for 15 minutes at 4°C, collect the supernatant, heat at 95°C for 8-15 minutes, followed by an ice bath for 2 minutes, then add an adequate amount of IAM solution and incubate in the dark for 1 hour. Then samples were completely mixed with 4 times volume of precooled acetone by vortexing and incubated at -20℃ for at least 30 min. Samples were then centrifuged at 12000 g for 15 min at 4℃ and the precipitation was collected. After washing with 1mL cold acetone, the pellet was dissolved completely by Dissolution Buffer (DB buffer).

1.2. Protein Quality Test

BSA standard protein solution was prepared according to the instructions of Bradford protein quantitative kit, with gradient concentration ranged from 0 to 0.5 μg/μL. BSA standard protein solutions and sample solutions with different dilution multiples were added into 96-well plate to fill up the volume to 20 μL, respectively. Each gradient was repeated three times. The plate was added 180 μL G250 dye solution quickly and placed at room temperature for 5 minutes, the absorbance at 595 nm was detected. The standard curve was drawn with the absorbance of standard protein solution and the protein concentration of the sample was calculated. 20 μg of the protein sample was loaded to 12% SDS-PAGE gel electrophoresis, wherein the concentrated gel was performed at 120 V for 20 min, and the separation gel was performed at 150 V for 50 min. The gel was stained by coomassie brilliant blue R-250 and decolored until the bands were visualized clearly.

1.3. Trypsin treatment [4]

Each protein sample was taken and the volume was made up to 100 μL with DB lysis buffer (6 M Urea, 100 mM TEAB, pH 8.5), trypsin and 100 mM TEAB buffer were added, sample was mixed and digested at 37 °C for 4 h. Formic acid was mixed with digested sample, adjusted pH under 3, and centrifuged at 12000 g for 5 min at room temperature. The supernatant was slowly loaded to the C18 desalting column, washed with washing buffer (0.1% formic acid, 3% acetonitrile) 3 times,

then added elution buffer (0.1% formic acid, 70% acetonitrile). The eluents of each sample were collected and lyophilized.

Table1 Liquid chromatography elution gradient table

Time	flow rate (μL/min)	mobile phase A (%)	mobile phase B（%）
0	2.5	96	4
0.2	1.3	96	4
0.3	0.8	92	8
0.5	0.8	92	8
14.2	0.8	77.5	22.5
21.1	0.8	65	35
21.5	2.5	45	55
21.5		Column Wash	
21.9	2.5	1	99
22.6	2.5	1	99
22.6	Stop Run		

1.4. LC-MS/MS Analysis-DIA mode

Prepare mobile phase A (99.9% water, 0.1% formic acid) and B (80% acetonitrile, 0.1% formic acid). The lyophilized powder was dissolved using 10μ LA solution, centrifuged at 14,000g for 20min at 4°C, and 200 ng of the supernatant sample was injected into the sample for liquid-quality detection. A Vanquish Neo upgraded UHPLC system was used with a C18 pre-column of 174500 (5 mm×300 μm ,5 μm, thermo) heated at 50°C in a column oven, and a C18 analytical column of ES906 (PepMap TM Neo UHPLC 150μm x 15 cm, 2 μm, thermo), and the elution conditions of the liquid chromatography were as shown in Table 1, a Thermo orbitrap astral mass spectrometer mass spectrometer was used, an Easy-spray (ESI) ion source was used, the ion spray voltage was set to 2.0 kV, the ion transfer tube temperature was set to 290°C, and the mass spectrum was in a dataindependent acquisition mode, with a full first-stage mass spectrometry scanning range of m/z 380-980. The primary MS resolution was set to 240000 (200m/z), AGC was set to 500%, the parent ion window size was set to 2-Th, the number of DIA windows was 300, the NCE was set to 25%, the secondary m/z acquisition range was from 150 to 2000, the sub-ion resolution Astral was set to 80000, and the maximal injection time was 3ms. Into mass spectrometry detection raw data (.raw).

1.5. Data analysis

1.5.1. The identification and quantitation of protein

The raw files were searched and analyzed using the DIA-NN library search software, according to the protein database. The mass deviation of precursor ions and fragment ions is automatically detected and corrected. The fixed modification is C carbamidomethylation, while N-term M excision is considered a variable modification. Up to 2 missed cleavage sites are allowed. To improve the quality of analysis results, the DIA-NN software further filters the search results, retaining only peptides with a Global.Q.Value < 0.01 and proteins with a PG.Q.Value < 0.01. A protein with a fold change (FC) greater than or less than a certain value (FC) was defined as a differentially expressed protein (DEP).

1.5.2. The functional analysis of protein and DEP

Gene Ontology (GO) and InterPro (IPR) functional analysis were conducted using the interproscan program against the non-redundant protein database (including Pfam, PRINTS, ProDom, SMART, ProSite, PANTHER)[5], and the databases of COG (Clusters of Orthologous Groups) and KEGG (Kyoto Encyclopedia of Genes and Genomes) were used to analyze the protein family and pathway. DEPs were used for Volcanic map analysis, cluster heat map analysis and enrichment analysis of GO, IPR and KEGG[6].

2. Transcriptomic Analysis

2.1 RNA Extraction and Library Preparation

Total RNA was extracted from cartilage tissue samples using TRIzol® reagent (Thermo Fisher Scientific, USA) according to the manufacturer's instructions. RNA integrity and quantity were verified using an Agilent Bioanalyzer (Agilent Technologies, USA). Libraries were prepared using the NEBNext® Ultra™ II Directional RNA Library Prep Kit (New England Biolabs, USA) following the manufacturer's protocol. Briefly, messenger RNA was purified from total RNA using poly-T oligo-attached magnetic beads. After fragmentation, the first strand cDNA was synthesized using random hexamer primers. The second strand cDNA was then synthesized using dUTP instead of dTTP, enabling strand specificity. The directional library was prepared after end repair, A-tailing, adapter ligation, size selection, USER enzyme digestion, amplification, and

purification. Library quality was checked with Qubit (Thermo Fisher Scientific, USA) and real-time PCR for quantification, and a bioanalyzer for size distribution detection.

2.2 Clustering and Sequencing

After library quality control, different libraries were pooled based on the effective concentration and targeted data amount, then subjected to Illumina NovaSeq 6000 platform sequencing (150 bp paired-end reads). The basic principle of sequencing is "Sequencing by Synthesis," where fluorescently labeled dNTPs, DNA polymerase, and adapter primers are added to the sequencing flow cell for amplification. As each sequencing cluster extends its complementary strand, the addition of each fluorescently labeled dNTP releases a corresponding fluorescence signal. The sequencer captures these fluorescence signals and converts them into sequencing peaks through computer software, thereby obtaining the sequence information of the target fragment.

2.3 Bioinformatics Analysis Pipeline

2.3.1 Data Quality Control

Raw data (fastq format) were initially processed using fastp software to obtain clean data by removing reads containing adapters, poly-N, and low-quality reads. The steps for data processing were as follows: (1) Discard a paired reads if either one read contains adapter contamination; (2) Discard a paired reads if more than 10% of bases are uncertain in either one read; (3) Discard a paired reads if the proportion of low quality (Phred quality <= 5) bases is over 50% in either one read. At the same time, Q20, Q30, and GC content of the clean data were calculated. All downstream analyses were based on the high-quality clean data.

2.3.2 Reads Mapping to the Reference Genome

Reference genome (GRCh38) and gene model annotation files were downloaded from a genome website. HISAT2 (v2.2.1) was used to build the index of the reference genome and to align paired -end clean reads to the reference genome. HISAT2 can use the gene model annotation file to create splice-aware alignments, providing better alignment accuracy compared to other non-splice alignment tools.

2.3.3 Novel Transcripts Prediction

The mapped reads of each sample were assembled by StringTie (v2.2.3) in a reference-based approach. StringTie uses a novel network flow algorithm as well as an optional de novo assembly step to assemble and quantitate full-length transcripts representing multiple splice variants for each gene locus.

2.3.4 Quantification of Gene Expression Level

FeatureCounts (v2.0.6) was used to count the reads numbers mapped to each gene. Then, FPKM (Fragments Per Kilobase of transcript sequence per Millions base pairs sequenced) of each gene was calculated based on the length of the gene and reads count mapped to this gene. FPKM considers the effect of sequencing depth and gene length for the reads count at the same time, and is currently a commonly used method for estimating gene expression levels.

2.3.5 Differential Expression Analysis

Differential expression analysis for two conditions/groups was performed using the DESeq2 R package (v1.42.0). DESeq2 provides statistical programs for determining differential expression in digital gene expression data using models based on negative binomial distribution. The resulting P-value was adjusted using the Benjamini and Hochberg's methods to control the false discovery rate. The threshold for significant differential expression was defined as $padj \leq 0.05$ and $|log2(foldchange)| \geq 1$.

2.3.6 GO and KEGG Enrichment Analysis of Differentially Expressed Genes

Gene Ontology (GO) enrichment analysis of differentially expressed genes was implemented by the clusterProfiler R package (v4.8.1), in which gene length bias was corrected. GO terms with corrected P-value less than 0.05 were considered significantly enriched by differentially expressed genes. KEGG (Kyoto Encyclopedia of Genes and Genomes) is a database resource for understanding high-level functions and utilities of the biological system. We used the clusterProfiler R package to test the statistical enrichment of differentially expressed genes in KEGG pathways.

Reference:

[1]Kachuk,Carolyn,Stephen,etal.Comparisonof sodiumdodecylsulfatedepletiontechniques forproteomeanalysisbymassspectrometry[J].Journalof chromatography, A: Including electrophoresis and other separation methods, 2015.

[2]Wiśniewski,JacekR,Zoμgman,etal.Universalsamplepreparationmethodforproteome analysis[J]. Nature Methods, 2009.

[3]SatpathyS, Mannan R, AnaRI.Proteogenomic Characterization RevealsTherapeuticVulnerabilities in LungAdenocarcinoma[J]. Cell, 2020,182(1).

[4]ZhangH,Liu T,Zhang Z,etal.Integrated ProteogenomicCharacterizationofHumanHigh- Grade Serous Ovarian Cancer[J]. Cell,2016.

[5] Huang D W,Sherman B T, LempickiR A.Bioinformaticsenrichment tools:pathstowardthecomprehensive functional analysis oflarge gene lists[J].NucleicAcids Research, 2009, 37(1):1-13.

[6]Franceschini A,SzklarczykD,FrankildS,etal.STRINGV9.1:Protein-ProteinInteraction Networks, with Increased Coverage and Integration[J]. NucleicAcids Research, 2012, 41(D1).
